# Stem Cells in Cartilage Regeneration

**DOI:** 10.1155/2017/7034726

**Published:** 2017-02-02

**Authors:** Jianying Zhang, Shiwu Dong, Wesley Sivak, Hui Bin Sun, Kai Tao

**Affiliations:** ^1^University of Pittsburgh School of Medicine, Pittsburgh, PA, USA; ^2^Third Military Medical University, Chongqing, China; ^3^Albert Einstein College of Medicine, New York, NY, USA; ^4^General Hospital of Shenyang Military Command, Shenyang, China

Cartilage is one of the chief structural tissues in the human body. Being resilient and smooth, it covers and protects the ends of long bones providing points of articulation at the joints and is a principal constituent of the rib cage, the ear, the nose, the bronchial tubes, the intervertebral discs, and many other body components [1]. It is not as hard and rigid as bone, but it is stiffer and less flexible than muscle.

Articular cartilage injuries in joints are frequently symptomatic and eventually lead to osteoarthritis, a disease reaching epidemic proportions within an increasingly aged population [2]. Osteoarthritis is a disease of the joint as an organ and is characterized by cartilage breakdown and alterations in the underlying subchondral bone [3]. It is estimated that about 27 million Americans aged 25 and older have osteoarthritis [4, 5]. Degeneration of the intervertebral disc, a fibrocartilaginous joint residing between adjacent vertebrae in the vertebral column, is the most frequent cause of low back pain and another significant cartilage-related disease [6]. The overall cost of chronic low back pain exceeds the combined costs of stroke, respiratory infection, diabetes, coronary artery disease, and rheumatoid disease [7].

Currently, a lack of understanding exists regarding the mechanisms that underly and connect the development of bone and cartilage diseases, resulting in few effective therapeutic options. For instance, there is poor angiogenic response of cartilage as a result of injury; therefore, damaged cartilage remains largely avascular and has poor regenerative potential [8]. Consequently, there is an urgent need to improve the understanding of the causes of bone and joint diseases and to identify new disease models to develop better treatments.

Stem cells play a vital role in tissue repair and regeneration in response to injury. The aim of this special issue is to understand the relationship between the stem cells and cartilage regeneration. Within this issue, C. Sang and colleagues isolated two stem cell populations from the nucleus pulposus (NP) and annulus fibrosis (AF) of rabbit intervertebral disc (IVD) and studied the differential properties of these two kinds of stem cells. This study demonstrates for the first time that the stem cells in NP and AF of rabbit IVD exhibit differential properties, which may prove useful for devising new biological approaches for regenerating damaged IVD in affected patients.

Mesenchymal stem cell- (MSC-) based therapy is regarded as a potential tissue engineering strategy to achieve nucleus pulposus (NP) regeneration for the treatment of intervertebral disc degeneration (IDD). However, it is still a challenge to induce MSC differentiation in NP-like cells when MSCs are implanted into the NP. Y. Gan et al. developed a controlled release codelivery system of MSCs encapsulated in dextran/gelatin hydrogel with TGF-*β*3-loaded nanoparticles for NP regeneration. They used poly(D, L-lactide-co-glycolide) (PLGA) nanoparticles as carriers for TGF-*β*3 and established a codelivery system of MSCs encapsulated in the dextran/gelatin hydrogel with TGF-*β*3-PLGA nanoparticles. In vitro studies showed that the codelivery system exhibited favorable cytocompatibility and the nanoparticles could reliably release active TGF-*β*3 over timeframes suitable to induce MSCs differentiation into NP-like cells while also promoting ECM-related biosynthesis. These results suggest this codelivery system may be employed as a promising strategy for discogenesis of MSCs in situ.

Also in the current issue, W. Fu et al. isolated and characterized mesenchymal stem cells (MSCs) from meniscal debris, while A. Hatakeyama and his colleagues compared MSCs isolated from the hip and knee synovium. Although the MSCs were derived from different articular tissues, they both can be differentiated into three lineages, namely, osteogenic, chondrogenic, and adipogenic. Furthermore, A. Hatakeyama et al. indicated that the knee synovium is a better source of MSCs compared to hip synovium.

The review article authored by J. Li and S. Dong provides an excellent summary of the signaling pathways involved in chondrocyte differentiation and hypertrophy. In this complex signaling network, key factors were outlined and discussed including bone morphogenetic proteins (BMPs), SRY-related high-mobility group gene 9 (Sox9), parathyroid hormone related peptide (PTHrP), Indian hedgehog (Ihh), and fibroblast growth factor receptor 3 (FGFR3). In addition, the role of reactive oxygen species (ROS) in cartilage formation was described. Better understanding of these signaling pathways will help researchers comprehend the regulatory mechanism of chondrogenesis, which is necessary in maintenance of normal chondrocyte phenotype.

The regulatory mechanism of the Ihh/PTHrP signaling pathway in fibrochondrocytes is further studied in a model of pig Achilles tendon by X. Han and his colleagues in this special issue. They treated the fibrochondrocytes of pig Achilles tendon with differential stress and found that the proliferation and differentiation of fibrochondrocytes are affected by cyclic stress tensile. The gene expression and protein synthesis of PTHrP, collagen I, and collagen II increased under low cyclic stress tensile. However, the gene expression and protein synthesis of Ihh and collagen X increased under high cyclic stress tensile. They conclude that proliferation and differentiation of fibrochondrocyte are regulated by stress stimulation through the Ihh and PTHrP signaling pathway.

Another review article authored by M. Wang et al. presents a summary of basic research using chondrocyte and stem cell technologies. An indepth discussion of the mechanisms of the damage and repair of cartilage provides several advanced strategies for the cartilage repair.

Autologous chondrocyte implantation (ACI) is a cell-based therapy that has been used clinically for over 20 years to treat cartilage injuries more efficiently in order to negate or delay the need for joint replacement surgery. However, the disadvantages of ACI such as cost, potential donor-site morbidity, and the quality of regenerated tissue are areas targeted for improvement [9]. In this special issue, J. Garcia et al. examined the chondrogenic potential of four different cell types from the patients with knee replacements including chondrocytes, bone marrow-derived mesenchymal stem cells (BM-MSCs), infrapatellar fat pad-derived MSCs (FP-MSCs), and subcutaneous fat derived MSCs (SCF-MSCs). They compared donor-matched cell types and established the impact of tissue source and donor on chondrogenic differentiation capacity. They demonstrated that there is a chondrogenic potency hierarchy ranging across these cell types, with the most potent being chondrocytes, followed by FP-MSCs, BM-MSCs, and lastly SCF-MSCs. Their findings have significant clinical implications for the refinement and development of novel cell-based cartilage repair strategies.

In addition, a coculture system was developed by J. Shi et al. that shows that the proliferation of chondrocytes was promoted and the apoptosis of the chondrocytes was inhibited when cocultured with adipose-derived stem cells (ADSCs). The similar results were obtained by adding TGF-*β*1 into culture medium, indicating that ADSCs may produce high concentrations of TGF-*β*1 to enhance the proliferation of chondrocytes. Furthermore, the authors found that the cocultured cells produced more anabolic proteins and inhibited catabolic proteins. These findings may provide a new solution for the treatment of cartilage injuries.

We hope this special issue provides a wealth of basic scientific data and introduces new concepts facilitating the development of advanced techniques and approaches for cartilage regeneration and repair.

*Jianying Zhang* *Shiwu Dong* *Wesley Sivak* *Hui Bin Sun* *Kai Tao*
